# Workplace Stress in Portuguese Oncology Nurses Delivering Palliative Care: A Pilot Study

**DOI:** 10.3390/nursrep12030059

**Published:** 2022-08-13

**Authors:** Cristina Costeira, Filipa Ventura, Nelson Pais, Paulo Santos-Costa, Maria Anjos Dixe, Ana Querido, Carlos Laranjeira

**Affiliations:** 1 School of Health Sciences of Polytechnic of Leiria, Campus 2, Morro do Lena, Alto do Vieiro, Apartado 4137, 2411-901 Leiria, Portugal; 2 Centre for Innovative Care and Health Technology (ciTechCare), Rua de Santo André—66–68, Campus 5, Polytechnic of Leiria, 2410-541 Leiria, Portugal; 3 The Health Sciences Research Unit: Nursing (UICISA: E), Nursing School of Coimbra (ESEnfC), 3004-011 Coimbra, Portugal; 4 Portuguese Oncologic Institute of Coimbra—Pain Unit, 3004-011 Coimbra, Portugal; 5 Center for Health Technology and Services Research (CINTESIS), NursID, University of Porto, 4200-450 Porto, Portugal; 6 Research in Education and Community Intervention (RECI I&D), Piaget Institute, 3515-776 Viseu, Portugal

**Keywords:** nurses, workplace stress, oncology, palliative care, nursing education

## Abstract

Oncology nurses often face complex end-of-life issues, underlining their need for specific training in palliative care. In this context, nurses experience several emotional and psychological dilemmas, which are often difficult to manage and result in high levels of workplace stress. This study aimed to determine the levels and work-related factors of workplace stress among oncology nurses. A descriptive baseline study was performed as part of a large four-phase study based on quantitative data collected from Portuguese oncology nurses. Of the 32 participating nurses, most were women, and the mean age was 42.69 ± 10.04 years. Overall, nurses revealed moderate levels of stress. Younger nurses with less professional experience had difficulties dealing with issues related to death and dying. This pilot study supported the development of a program of six Stress Management Training Workshops (SMTW) to reduce stress and increase adaptative strategies. Assessing workplace stress among oncology nurses should be the focus of intervention by managers and institutional leaders.

## 1. Introduction

Nurse inability to meet work demands can lead to illness or psychological distress. The literature indicates elevated stress levels among nurses, partly due to the current complexity of clinical environments [1,2,3]. Studies indicate that workplace stress is an alarming worldwide phenomenon [4,5]. The global prevalence of workplace stress among nurses was reported to be approximately 9–68%, varying across different geographical, cultural and clinical settings [6]. Workplace stress has been linked to poor performance, absenteeism, high staff turnover, and low job satisfaction [1,7,8,9,10], as well as physical and psychological illnesses, such as increased rates of substance abuse [11], anxiety and depression [12], insomnia [13], other psychological disorders [14], burnout [3], and lifetime rates of suicidal ideation [15]. Workplace stress can also lead to unhealthy coping strategies such as smoking and other maladaptive behaviors [16].

Studies of workplace stress have centered on how it is generated, focusing on two major sets of causes. The first includes how stressful psychosocial characteristics of work settings, such as increasing workloads, role conflict, lack of autonomy, and lack of social support, might contribute to workplace stress and impede performance [17]. The second encompasses environmental elements, namely how workers’ abilities and their physical surroundings affect performance and whether a person–environment “misfit” leads to unfavorable psychological or physiological reactions [18]. However, there is another key factor that can cause stress in employees: ambiguity and potentially dangerous circumstances in the workplace. As a result, components of the physical environment can raise unnecessary expectations among workers or interfere with their capacity to perform, thereby causing stress and hindering their performance [17].

The act of identifying and applying techniques or interventions to decrease or regulate workplace stress is known as stress management [19], which includes coping strategies [20]. Nurses must learn and practice effective stress management strategies to help them better adapt to work-related stress [21]. Two main types of stress reduction interventions are found in the nursing literature. The first focuses on reducing stressors by improving corporate practices, procedures, and organizational culture [22]. The second focuses on implementing cognitive-behavioral interventions (CBIs), such as cognitive-behavioral therapy, meditation, and relaxation response, which have been demonstrated to lower stress in nurses [23]. Because stressors can be the direct result of job pressures or associated personal demands, a mixture of stress reduction measures related to both the work and personal environment is beneficial [24].

### 1.1. Theoretical Framework

A socioecological approach portrays workplaces as adaptive systems composed of personal, social, technological, and organizational components that interact in non-linear ways [25]. The System Dynamics Model (SDM) of workplace stress, developed by Jetha et al. [25], considers a wide range of components, such as organizational environment, workplace social support, workplace safety, and work–family conflict. The SDM captures the cyclical nature of workplace stress, viewed as an effect and predictor of situations, both inside and outside the workplace. Accordingly, workplace stress may be addressed by implementing workplace interventions that consider an organizational system’s numerous aspects and the feedback links between model components [25].

### 1.2. Research Problem

As mentioned above, workplace stress manifests itself in several ways, with personal and organizational impacts on workplace performance, resulting in absenteeism, job turnover, a negative organizational atmosphere, and lower productivity [26]. To meet the physical and emotional demands of their job, nurses must be free of workplace stress and maintain excellent health. Few studies have explored this topic in Portugal, particularly in oncology settings. Since oncology nurses care for people and families with palliative needs, they have an increased risk of workplace stress, due to the psychological and emotional exhaustion and suffering they face when dealing with patients and families [27,28,29,30,31]. To best manage stress symptoms and increase overall mental well-being [32], nurses need information, advocacy, training, programming, and resources in the areas of positive coping and self-care. Additionally, the COVID-19 pandemic crisis increased the daily pressure and difficulties these professionals face in managing stress [26].

Therefore, the main aim of this study was to determine the levels and work-related factors of workplace stress among oncology nurses working in a Portuguese Oncology Hospital. This study’s research questions were: (a) What are the stress levels of nurses in an oncology care setting? and (b) What work-related factors influence workplace stress levels? This empirical information is critical for recommendations on how to develop Stress Management Training Workshops (SMTW) for nurses.

## 2. Materials and Methods

### 2.1. Study Design

**Figure 1:**
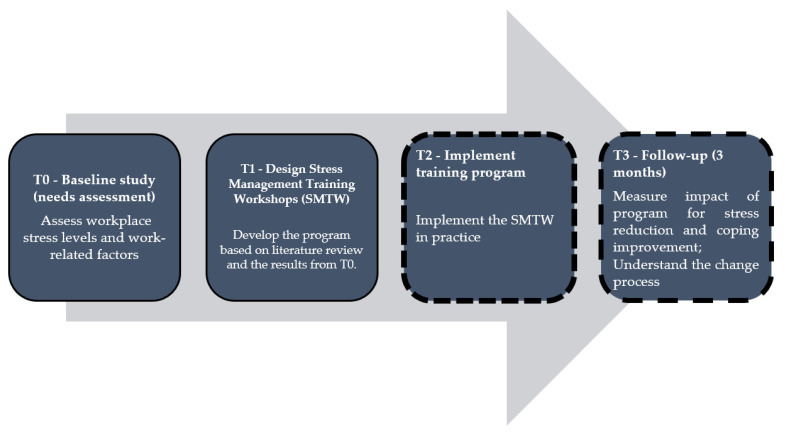
Overview of study phases and objectives.

A descriptive baseline study was performed as part of a larger study composed of four phases (Figure 1) and guided by the multidimensional System Dynamics Model (SDM) of workplace stress [25]. The present study (T0) was based on quantitative data (collected in the last months of 2021) and supported the development of a SMTW (T1) for oncology nurses to reduce their stress and increase adaptative strategies. The Strengthening the Reporting of Observational Studies in Epidemiology (STROBE) guidelines were followed.

### 2.2. Sample Recruitment

Participants were recruited from one Portuguese Oncology Hospital in the central region of Portugal. Nurses with a bachelor’s degree or higher, delivering care to palliative patients/families, and working as full-time staff for at least 6 months were eligible for inclusion in this study. Individuals were excluded if they refused to participate in this study or did not complete the questionnaires.

### 2.3. Data Collection

Participants were contacted by email through institutional mailing lists, briefed about the study goals, and encouraged to participate. The online survey was distributed through email by the principal investigator to 65 eligible nurses. Returning the completed questionnaire implied that consent had been provided. The response rate was 49.23%, with a final sample of 32 nurses.

Data were collected using a self-reporting questionnaire consisting of two parts: (I) sociodemographic and professional data; (II) Nurse Workplace Stress Scale (NWSS) [33]. The NWSS is a 4-point Likert scale, ranging from 1—never to 4—very often, and composed of 34 items, with no reversed items. These are grouped into seven factors: “death and dying”, “conflict with doctors”, “inadequate preparation to deal with the emotional needs of patients and family members”, “lack of support from colleagues”, “conflicts with other nurses and bosses”, “workload” and “uncertainty about treatments”.

Global scores ranged from 34 to 136, with higher values corresponding to severe levels of nurse workplace stress. Internal consistency values were calculated for the current sample using Cronbach’s alpha for the total scale and each factor; there were significant values of internal consistency (∝ = 0.97 for the total scale). Satisfactory results were also found for each scale factor: factor I “death and dying” ∝ = 0.86; factor II “conflict with doctors” ∝ = 0.88; factor III “inadequate preparation to deal with the emotional needs of patients and family members” ∝ = 0.86; factor IV “lack of support from colleagues” ∝ = 0.79; factor V “conflicts with other peers and nurse managers” ∝ = 0.81; factor VI “workload” ∝ = 0.86; and factor VII “uncertainty about treatments” ∝ = 0.91.

### 2.4. Data Analysis

Descriptive and inferential statistics were used. Frequencies, percentages, medians, means and standard deviations were used for sample description. Since the data were not normally distributed, non-parametric tests were performed. The Spearman rank correlation was used to evaluate the degree of correlation between quantitative variables. The Mann–Whitney U test was used to compare differences between two independent groups. Data were analyzed using SPSS-26 at a significance level of 0.05.

### 2.5. Ethical Procedures

Participation implied consent. Participant anonymity was preserved, and the researchers were blinded by employing a web-based survey application to gather and collate data. Non-responders were automatically detected by the computerized system, and reminder emails were only sent to subscribers who had not answered the prior email(s). Multiple replies from the same Internet Protocol (IP) address were rejected by the Google Form settings. This study was reviewed and approved by the Oncology Hospital’s Ethical Committee (TI no 08/2021).

## 3. Results

### 3.1. Sample Characteristics

Of the 32 respondents, most were women (*n* = 27) and the mean age was 42.69 ± 10.04 years (range 35–60). The average professional experience was 19.56 ± 9.80 years (range 3–35), and the average time working in the current oncology ward was 19.34 ± 9.98 (range 4–35). In this group, only 15.6% (*n* = 5) of professionals reported having basic training in palliative care.

### 3.2. Nurse Workplace Stress

Analysis of all 34 items suggests nurses presented significant workplace stress, with average scores above two, the same being true for all factors in the scale (Table 1). Factors “Death and dying”, “Workload” and “Uncertainty with treatments” presented a higher average score, indicating higher workplace stress.

A global analysis of the scale indicated there was a statistically significant difference between nurses with training versus nurses with no training in palliative care (U = 18.00; *p* = 0.01). Trained nurses had higher stress scores (Md = 3.59; M = 3.37; SD = 0.54) compared to nurses without training in palliative care (Md = 2.35; M = 2.53; SD = 0.54). There were no significant differences between male and female nurses, in any factor or item.

#### 3.2.1. The Intrapersonal Domain of Nurse Workplace Stress

Table 2 presents the results of three intrapersonal factors related to self-knowledge in specific situations/contexts, namely: Factor I—Death and dying; Factor II—Inadequate preparation to deal with the emotional needs of patients and their families; and Factor III—Uncertainty with treatments.

The findings show that age and professional experience was negatively correlated with factor I (issues related to death and dying) and its item 4 (the feeling of powerlessness when a patient does not improve with treatment). On the other hand, novice nurses presented higher levels of stress than older nurses and those with more professional experience.

We noticed significant differences between nurses with and without training in palliative care in the three factors related with intrapersonal issues (Table 2). These results indicate that nurses trained in palliative care—situations demanding higher quality of care for the patient—presented higher stress values when these conditions were not guaranteed.

#### 3.2.2. The Interpersonal Domain of Nurse Workplace Stress

In Table 3, three factors related to interpersonal relations are grouped, namely: “Conflicts with doctors”; “Lack of support from colleagues” and “Conflicts with other peers and nurse managers”. There were statistically significant differences between nurses with and without training regarding levels of stress related to conflicts with doctors or superiors, when they are criticized by them, when there are disagreements about the patient’s treatment, when they feel a lack of support from colleagues, and when they are mobilized to other services. The results show that interpersonal conflicts and lack of support make nurses trained in palliative care more susceptible to stress.

#### 3.2.3. The Organizational Domain of Nurse Workplace Stress

Table 4 presents the organizational domain of the workplace stress scale, which refers to the “Workload” factor. Stress values related to workload, lack of time and lack of staff presented statistically significant differences, with nurses trained in palliative care showing higher levels of stress.

## 4. Discussion

In this paper, we have presented a workplace stress assessment tool for oncology nurses and have systematized a stress management intervention program to fill a gap in oncology nursing education. Nurses are considered a professional occupation with high risk of workplace stress [27,28,30,31]. Our data indicate that nurses had a moderate level of workplace stress, exposing these professionals to various personal consequences and impacting the quality and safety of care provided [2,7,8,9,10].

Our findings suggest that novice nurses, with less professional experience in caring for people with palliative needs, have higher levels of stress associated with “Death and dying”. Similar results were found by Kim and Kim [30] and Zheng et al. [34], who also showed that more experienced nurses have more effective stress management strategies. Our findings also suggest that nurses with training in palliative care have higher values of workplace stress. In contrast, in a similar sample, Pais et al. [29] showed that training in palliative care facilitates emotional regulation in the face of death, reducing the associated stress. This disparity can be explained by the fact that nurses in our study only had basic training in palliative care, typically oriented towards the patient and family care and less focused on the needs of health professionals. Nevertheless, the importance of specific training in palliative care is valued internationally, as only through the training of health professionals will they be able to obtain and guarantee the quality and safety of care [35].

While individual-group interventions are beneficial for promoting personal mental health, health care organizations must acknowledge that changes at the managerial, team, cultural, and organizational levels are essential to ensure the effectiveness of treatments [36,37]. On this point, Milig et al. [38] emphasized the need for nursing managers to develop activities that help reduce nurse stress and minimize negative consequences associated with stressful professional contexts and environments.

The structure of current training programs in palliative care should be reconsidered in this context. Previous interventions in workplace stress management recommended the development of communication skills [39], emotional competence [40,41], spiritual needs [42] and practical stress management strategies [43,44,45,46,47,48,49]. The areas recommended by these studies are consensual in the development of training programs in palliative care.

Some recommendations refer to the need to promote active educational modalities, through the training of communication and interpersonal skills using simulation practices, digital interventions, and establishing partnerships between palliative care services and training institutions, all important aspects to be addressed in educational intervention programs [50,51,52]. Our findings prioritize some training needs, such as intra and interdisciplinary conflicts, the difficulty in dealing with death and dying processes, the management of complex care situations and the relationship with patients and families. Overall, our study’s findings provide new knowledge to enhance palliative care practice and education. Self-care is a proactive and personalized approach to promote health and wellness that uses a range of tactics in both intimate and professional contexts to improve the capacity for compassionate care among patients and their families.

### 4.1. Contributions to the Development of Stress Management Training Workshops (SMTW)

The SMTW is an experiential learning program consisting of a cognitive-behavior intervention centered on stress management strategies for nurses. Experiential learning is an engaged learning process whereby participants “learn by doing” and by reflecting on the experience [53]. The SMTW was administered by a specialized mental health nurse (i.e., specialize in stress management), and consisted of six weekly 90 min group sessions, each based on the change objectives (Table 5). As a multidimensional approach to improve coping and well-being inside and outside the workplace, SMTW was designed to promote growth in many areas of well-being including emotional, psychological, spiritual, and social [32]. The workshop schedule was reviewed by the nurse manager, based on staff availability, and conducted outside the scope of daily duties. Evidence suggests that small groups, with 8–10 participants [32], provide the most conducive environment for participants to benefit from the program. Based on the results obtained and given the potential for sharing generated around the theme of stress, the groups should be multigenerational. More experienced nurses will be able to share their experiences and thus facilitate the acquisition of adaptive mechanisms by novice nurses.

All sessions must use interactive learning styles, such as lectures, class discussion, role-playing sessions with case scenarios, thus facilitating the learning process and enhancing retention of information. Furthermore, to allow each participant to implement techniques, the demonstration and re-demonstration teaching approach must be employed.

The atmosphere of the workshops should be one of empathy, validation, and respect, with a focus on the importance of maintaining dialogue and keeping communication channels open. The sessions should be concluded with a debriefing, since this is a key approach for learning about and improving individual, team, and organizational performance. Debriefing, whether formal or informal, is frequently described as an effective self-care method and should thus be promoted [61].

### 4.2. Study Strengths and Limitations

Our study has some strengths. First, the provision of tailored interventions should include individual assessment of the target-intervention groups, as performed in the present study. Second, when constructing the intervention, empirical data and stress management literature was employed to create a solid basis for achieving the change objectives. Third, the intervention program was brief, with only six sessions, to provide brief, feasible, and effective interventions. Finally, the interventions and assignments in this program were designed and widely used, which we believe increases the program’s validity.

The interpretation of the results might be limited by some study specificities. First, this study was carried out in a single hospital with a relatively small number of questionnaires. The voluntary recruitment strategy could explain the strong non-response bias. Second, expanding data collection to other oncological settings would deepen the understanding of ‘workplace stress’ in relation to contextual heterogeneity, thereby enhancing the program’s external validity. Furthermore, the program’s impact has not yet been ascertained. Lastly, the intervention’s effectiveness may depend on the skill, experience and style of the professional performing the intervention.

### 4.3. Implications for Practice

This study has implications for clinical practice and education and health organizations. Knowledge of workplace stress levels among palliative care nurses allows the anticipation and adaptation of strategies and tools towards managing high levels of workplace stress. Such endeavors are likely to avoid negative consequences for the mental and physical health of professionals and indirectly contribute to improve the health care provided to the patient and family. This study can also be used to alert health managers, decision-makers and entities responsible for nurse training to the need for improving current working conditions, thereby encouraging strategies aimed at managing workplace stress and the inclusion in curricular contents of basic training in palliative care. Thus, a reduction in the economic impact associated with high levels of stress is anticipated, particularly in work phenomena such as absenteeism, presenteeism and turnover.

## 5. Conclusions

Recognizing that nurse workplace stress is indicative of well-being and mental health status, nurses with high levels of stress tend to develop a flawed person-centered practice in a palliative situation for patients and their families, often adopting escape and avoidance strategies. Here, the role of health managers and decision-makers is crucial as they can mediate stress levels. In particular, health managers may promote programs to support effective stress management, where factors such as the age of professionals, professional experience and training in palliative care should be taken into account. Curricula of basic training in palliative care should be rethought, privileging active strategies for training and personal development in palliative care, so that professionals acquire theoretical knowledge and are able to mobilize and implement it. The development of intervention strategies that improve nurses’ skills in situations related to death and dying must also be considered in the effective management of workplace stress in nurses.

## Figures and Tables

**Table 1:** Descriptive statistics of Nurse Workplace Stress Scale.

	M	SD	Md	Percentile 25	Percentile 75
**Factor I—Death and dying**	2.89	0.66	2.71	2.29	3.75
**Factor II—Conflicts with doctors**	2.54	0.75	2.40	2.00	3.00
**Factor III—Inadequate preparation to deal with the emotional needs of patients and their families**	2.74	0.81	2.67	2.00	3.58
**Factor IV—Lack of support from colleagues**	2.63	0.82	2.50	2.00	3.33
**Factor V—Conflicts with other peers and nurse managers**	2.55	0.73	2.40	2.40	3.20
**Factor VI—Workload**	2.87	0.68	2.83	2.33	3.58
**Factor VIII—Uncertainty with treatments**	2.88	0.79	2.60	2.20	3.90
**Global score**	2.66	0.62	2.41	2.26	3.35

**Table 2:** Statistical analysis between intrapersonal domain and demographic and professional variables.

		Age	Current Service Experience	Years of Profession	Training in Palliative Care			
		rs	rs	rs	U	p	With Training	No Training
							**Md**	**Md**
**Factor I: Death and dying**		−0.52 **	−0.51 **	−0.50 **	27.00	0.04	3.57	2.71
items	3. Perform procedures that patients feel as painful	−0.48 **	−0.46 **	−0.48 **	19.00	0.01	4.00	3.00
	4. Feeling powerless when a patient does not improve with treatments	−0.62 **	−0.62 **	−0.61 **	24.00	0.02	4.00	3.00
	6. Talking to the patient about the proximity of death	−0.41 *	−0.43 *	−0.42 *	33.00	0.06	3.00	2.00
	8. The death of a patient	−0.15	−0.15	−0.12	50.50	0.34	3.00	2.00
	12. The death of a patient with whom a close relationship has developed	−0.42 *	−0.40 *	−0.39 *	39.50	0.13	3.00	2.00
	13. Absence of the doctor when a patient dies	−0.29	−0.29	−0.28	48.50	0.31	4.00	3.00
	21. Seeing a sick person in pain	−0.47 **	−0.47 **	−0.45 *	39.00	0.10	4.00	3.00
**Factor III: Inadequate preparation to deal with the emotional needs of patients and their families**		−0.33	−0.33	−0.31	16.00	0.01	3.67	2.33
items	15. Lack of preparation to support the patient’s family in their emotional needs	−0.25	−0.23	−0.24	32.50	0.06	4.00	3.00
	18. Not having an adequate answer to a question posed by the patient	−0.28	−0.29	−0.271	18.00	0.01	4.00	2.00
	23. Feeling unprepared to support the patient’s emotional needs	−0.31	−0.31	−0.29	19.00	0.01	4.00	2.00
**Factor VII: Uncertainty about treatments**		−0.43 *	−0.44 *	−0.43 *	27.00	0.03	4.00	2.60
items	17. Inadequate information provided by the physician regarding the patient’s clinical situation	−0.47 **	−0.474 **	−0.46 **	34.50	0.07	4.00	3.00
	26. Medical prescriptions apparently inappropriate for the treatment of a patient	−0.34	−0.364 *	−0.34	19.00	0.01	4.00	2.00
	31. Absence of a doctor during a medical emergency	−0.47 **	−0.472 **	−0.47 **	29.00	0.03	4.00	2.00
	32. Not knowing what to say to the patient and family about their condition and treatment	−0.31	−0.304	−0.29	40.50	0.14	4.00	3.00
	33. Doubts regarding the operation of certain specialized equipment	−0.45 *	−0.444 *	−0.44 *	35.00	0.06	4.00	2.00

rs—Spearman rank correlation; * p = 0.05; ** p = 0.001; U—Mann–Whitney U; p—significance level; Md—Median.

**Table 3:** Statistical analysis between interpersonal domain and demographic and professional variables.

		Age	Current Service Experience	Years of Profession	Training in Palliative Care			
		rs	rs	rs	U	p	With Training	No Training
							**Md**	**Md**
**Factor II: Conflicts with doctors**		−0.33	−0.37 *	−0.33	10.00	0.00	3.80	2.20
items	2. Being criticized by a doctor	−0.36 *	−0.41 *	−0.37 *	7.00	0.00	4.00	2.00
	9. Conflict with a doctor	−0.36 *	−0.39 *	−0.35 *	9.000	0.00	4.00	2.00
	10. Fear of making mistakes when treating a patient	−0.21	−0.27	−0.22	25.50	0.02	4.00	2.00
	14. Disagreement regarding the treatment of a patient	−0.26	−0.29	−0.27	26.00	0.02	4.00	2.00
	19. Making a decision regarding the patient’s treatment	0.12	0.10	0.13	36.00	0.07	3.00	2.00
**Factor IV: Lack of peer support**		−0.33	−0.33	−0.31	16.00	0.01	3.33	2.33
Items	7. Lack of opportunity to speak openly with other team members about service	−0.14	−0.18	−0.15	42.50	0.18	3.00	3.00
	11. Lack of opportunity to share experiences and feelings with other team members	−0.07	−0.04	−0.01	62.50	0.79	3.00	2.00
	16. Lack of opportunity to express negative feelings about the patient to other team members	−0.36 *	−0.35 *	−0.33	36.00	0.08	4.00	2.00
**Factor V: Conflicts with other nurses and bosses**		−0.21	−0.21	−0.18	46.50	0.27	3.40	2.20
items	5. Conflict with a superior	−0.55 **	−0.55 **	−0.53 **	24.50	0.02	4.00	2.00
	20. Being mobilized to another service to make up for staff shortages	−0.59 **	−0.58 **	−0.58 **	25.00	0.02	4.00	2.00
	22. Difficulty working with a particular nurse (or nurses) from another service	−0.04	−0.08	−0.05	53.00	0.41	2.00	2.00
	24. Receiving criticism from a superior	−0.39 *	−0.42 *	−0.39 *	7.500	0.00	4.00	2.00
	29. Difficulty working with a particular nurse (or nurses) from the same service	−0.17	−0.23	−0.19	56.00	0.52	3.00	2.00

rs—Spearman rank correlation; * p = 0.05; ** p = 0.001; U—Mann–Whitney U; p—significance level; Md—Median.

**Table 4:** Statistical analysis between organizational domain and demographic and professional variables.

		Age	Current Service Experience	Years of Profession	Training in Palliative Care			
		rs	rs	rs	U	rs	With Training	No Training
							**Md**	**Md**
**Factor VI: Workload**		−0.35 *	−0.38 *	−0.36 *	24.00	0.02	4.00	2.00
Items	1. Computer malfunction	−0.23	−0.24	−0.24	42.00	0.13	4.00	2.00
	25. Unexpected changes to the schedule and work plan	−0.45 *	−0.47 **	−0.45 *	34.50	0.07	4.00	3.00
	27. Too many tasks outside the strict professional scope, such as administrative work	−0.01	−0.03	−0.00	52.00	0.39	4.00	3.00
	28. Lack of time to give emotional support to the patient	−0.29	−0.32	−0.29	28.00	0.03	4.00	3.00
	30. Lack of time to perform all nursing activities	−0.37 *	−0.39 *	−0.37 *	38.00	0.10	4.00	3.00
	34. Lack of personnel to adequately cover the service needs	−0.45 **	−0.46 **	−0.45 **	33.00	0.05	4.00	2.00

rs—Spearman rank correlation; * p = 0.05; ** p = 0.001; U—Mann–Whitney U; p—significance level; Md—Median.

**Table 5:** The matrix of change objectives in the SMTW.

Session	Assignments	Change Objectives
Workshop 1	Psychoeducation session (definition, causes, and consequences of social and occupational life, as well as stress management styles and strategies); a psychoeducation pamphlet should be produced.	This educational approach increases knowledge and learning about the underlying causes of mental health problems and which evidence-based interventions can be used to treat such issues [32]. The pamphlet provides advice on finding indicators of stress and on relaxation and sleep hygiene, as well as stress reduction strategies such as exercise, laughter, and connecting with close friends.
Workshop 2	Relaxation techniques training (1—Deep Breathing Exercise; 2—Progressive Relaxation Techniques; and 3—Body scan, mindfulness meditation, self-compassion techniques).	By encouraging non-judgmental self-awareness and self-care, the participant gains a more stable and positive sense of self. The individual uses abilities to defend oneself, manage resources, and comfort oneself and others [54,55]. Emotion identification, self-validation, acknowledging and reframing self-critical thoughts, mindfulness meditation, and affirmations are all examples of self-compassion abilities [32].
Workshop 3	Positive self-talk and problem-solving skills are part of the cognitive restructuring techniques developed in this session.	This assignment focuses on recognizing misunderstandings, influencing skewed thinking, and thus reducing anxiety and boosting problem-solving abilities and reasoned practice [56].
Workshop 4	Humor therapy, in which participants practice humor through several methods (laughing videos, laughter meditation, sharing personal tales and jokes, and exchanging joyful ideas).	The participant improves emotion management by visual and linguistic expression of emotions, externalizing these feelings. In the nursing context, humor increases communication, well-being, and positive affect [57]. Individuals convey how they feel by expressing emotions and handling things in a constructive/helpful, comforting, and calming manner.
Workshop 5	Assertiveness training and time management.	The participant gains communication skills in the development and maintenance of strong interpersonal connections at work, as well as successful team functioning [58]. Individuals should leave this workshop with the necessary skills and information to speak more confidently and successfully, by employing assertive behavior tactics.This assignment also focuses on time management skills such as preparing lists, scheduling tasks, checking off each activity as it is completed, and avoiding distractions [59].
Workshop 6	Self-care training in facing death and dying—spirituality care.	Self-care reflexive individual exercises or in small groups, with different instruments (e.g., reflexive writing, visual aids, or storytelling).Topics discuss and reflect on personal values, including spirituality, being present during pain, the concept of dignity, spiritual self-care, hope, death, and the afterlife [60].

## Data Availability

The data are available upon reasonable request.
